# Estradiol shows anti-skin cancer activities through decreasing MDM2 expression

**DOI:** 10.18632/oncotarget.14275

**Published:** 2016-12-27

**Authors:** Li Li, Jianguo Feng, Ying Chen, Shun Li, Mengting Ou, Weichao Sun, Liling Tang

**Affiliations:** ^1^ Key Laboratory of Biorheological Science and Technology, Ministry of Education, College of Bioengineering, Chongqing University, Chongqing, China; ^2^ Department of Anesthesiology, The Affiliated Hospital of Southwest Medical University, Sichuan, China

**Keywords:** MDM2, hnRNPA1, estradiol, melanomas, therapeutic

## Abstract

Estradiol plays important roles in many biological responses inducing tumor genesis and cancer treatment. However, the effects of estradiol on tumors were inconsistent among a lot of researches and the mechanism is not fully understood. Our previous study indicated that splicing factor hnRNPA1 could bind to the human homologue of mouse double minute (MDM2), an oncogene which has been observed to be over-expressed in numerous types of cancers. In this research, we investigated whether and how estradiol correlate to cancer cell behaviors through heterogeneous nuclear ribonucleoprotein (hnRNPA1) and MDM2. Results showed that 10×10^-13^Mestradiol elevated the expression of hnRNPA1 regardless ER expression in cells, and then down-regulated the expression of MDM2. At the same time, estradiol inhibited cell proliferation, migration and epithelial-mesenchymal transition progression of A375 and GLL19 cells. While, knocking down hnRNPA1 through the transfection of hnRNPA1 siRNA led to the increase of MDM2 at both protein level and gene level *In vivo* experiment, subcutaneous injection with estradiol every two days near the tumor at doses of 2.5mg/kg/d suppressed tumor growth and reduced MDM2 expression. In a word, via increasing hnRNPA1 level and then reducing the expression of MDM2, estradiol prevented carcinogenesis in melanomas. We confirmed therapeutic effect of estradiol, as well as a new way for estradiol to resist skin cancer.

## INTRODUCTION

Estradiol is the predominantly estrogen in blood circulation of people [1]. As one kind of sex steroid hormones, it can balance the proliferation and differentiation of cells. Itis very essential to biological responses in physiology of brain, ovulation, and uterus [2–8]. The classic genomic model of estradiol is mediating transcription of several genes in cell growth by nuclear receptors such as ERα and ERβ [9]. Whereas, nogenomic correlations for estradiol through some intracellular signaling had also been revealed [10]. A wide number of reports have shown that estradiol has a tumor restrictive role in several cancer cell lines. For example, in MCF7 breast cancer cells, the anticancer evaluations of estradiol were shown through its anti-proliferative activity [11]. In the meanwhile, in B16F10 melanoma cells, although estradiol could not inhibit migration of these cells, it had inhibitory effect on invasiveness [11, 12]. What's more, in Ch27 and H1355 lung cancer cells, estradiol elevated P53 and P21 expression on protein level [5]. In these cancer cell lines, estradiol acts as a factor inhibiting tumor progression via a highly complex but not clear signaling system.

The mouse double minute-2 gene (MDM2) is an oncogenic gene that is overexpressed in various types of cancers including melanoma [13–15]. It is well-known that the alternative splicing of genes is frequently associated with the tumorigenic phenotype. For instance, the full-length MDM2, which was also an E3 ubiquitin ligase, bound and ubiquitylated tumor suppressor P53 leading to its degradation [16–20]. While MDM2^alt1^, one of the common variants of MDM2, has been detected to elevate the accumulation of mutant P53 and to exhibit anticancer activity [21]. Consistent with this, we want to discover a new strategy which can down-regulate the expression of MDM2 in skin-cancer cells via the alternative splicing of MDM2.

Arginine/serine-rich proteins and heterogeneous nuclear ribonucleoprotein (hnRNPA) can elevate diversity of the proteome, and they also bind the pre-mRNA splicing of multiple genes [6, 22]. Numerous reports have shown that hnRNPA1 has a good relationship with gene alternative splicing [23–25]. According to the result from *Donev*, estradiol could elevate the expression of hnRNPA1 in NT2N cells [15]. In addition, the up-regulation of hnRNPA1 and alternative splicing effect on many types of oncogenic genes had been tightly associated with the proliferation or motility of cancer cells [24, 26, 27]. Furthermore, hnRNPA1 was found to act as a UV-induced splicing factor for MDM2 in the previous study of our lab [28]. From this point onwards, we desire to focus on the estradiol-induced hnRNPA1 which can decrease MDM2 expression. This novel pathway may help us to find an efficient mechanism which contributes to cancer therapy.

Our data demonstrated that estradiol prevented proliferation, migration and EMT progression of melanomas *in vitro* by increasing the expression of hnRNPA1 and then influencing the level of MDM2 in melanomas. Injection of nude mice with estradiol led to the regression of tumors *in vivo*. These results are particularly important because the estradiol might become a new class of therapeutic agent for melanomas. Endocrine treatment should become an adjuvant therapy for hormone-dependent cancer.

## RESULTS

### Roles of estadiol in expressions of hnRNPA1 and MDM2 in different cells

**Figure 1:**
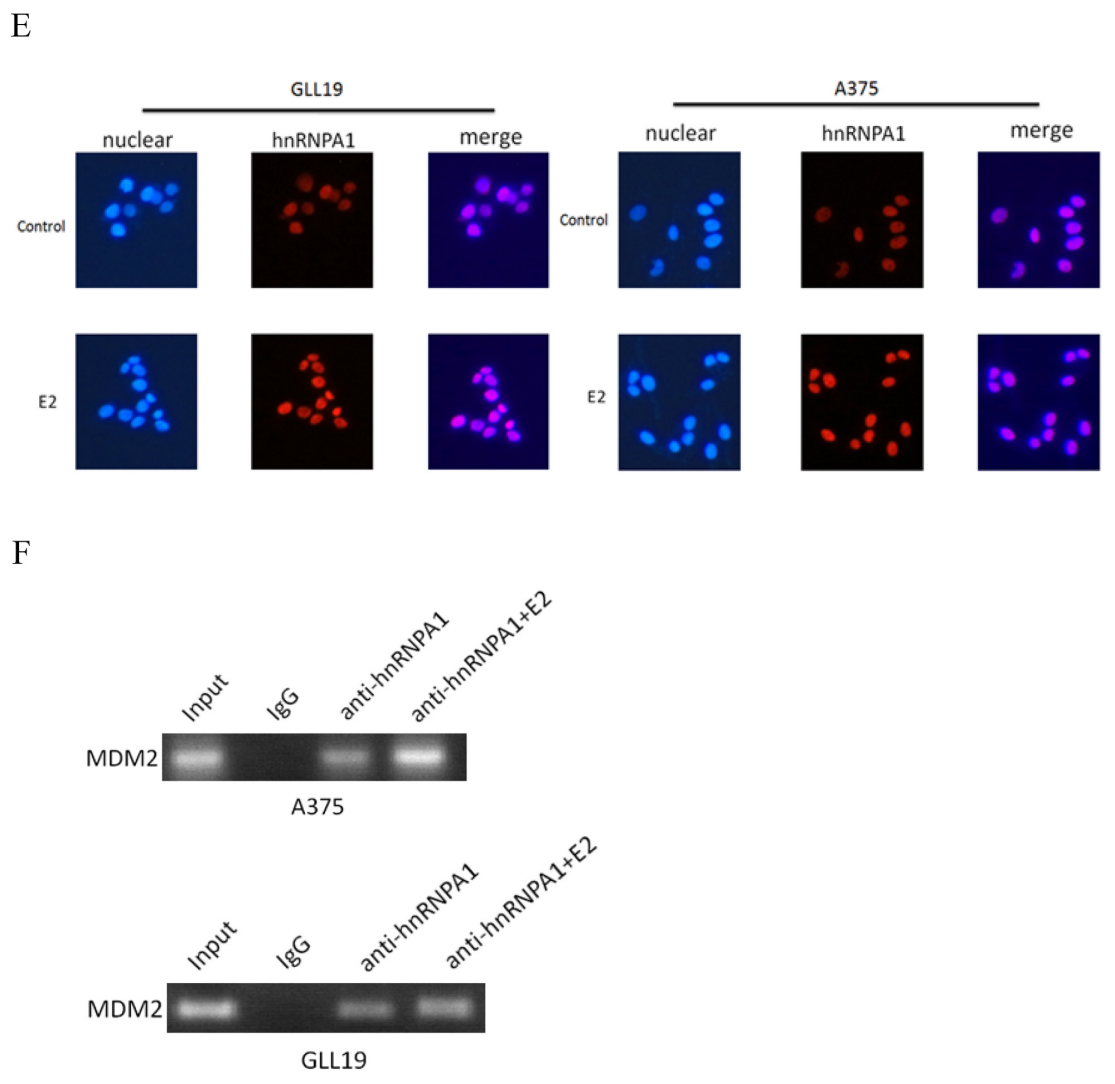
Estradiol-treatment results in the alteration of hnRNPA1 and MDM2 may not correlate to P53 Hacat and GLL19 cells were exposed to different concentrations of estradiol for96 hours for the expression of hnRNPA1 **A.** and cell proliferation **B.** (n=5, a representative experiment is shown; ‘*’means P<0.05;‘**’means P<0.01) Hacat, GLL19 and A375 cells were treated with or without 10×10-13M estradiol for 96 hours and evaluated the hnRNPA1, MDM2 and P53 expression at mRNA level. **C.** and at protein level **D. E.** GLL19 and A375 cells were treated with 10×10-13 M estradiol or vehicle for 96 hours, and then immunofluorescence detection was used. Nuclei were stained with DAPI and acted as internal references. **F.** the same number of cells between control and treatment group were harvested, and then RNA immunoprecipitation (RIP) was used to assay hnRNPA1-MDM2 binding. Results of gene and protein level were expressed as percent of GAPDH vs. vehicle-treated cells. (n=3, a representative experiment is shown; ‘*’means P<0.05; ‘**’means P<0.01).

GLL19 and Hacat cells were treated with different concentrations of estradiol from 10×10^-9^M to 10×10^-13^M. The expression of hnRNPA1 and cell proliferation was determined by RT-PCR and MTT assay, respectively. As shown in Figure 1, estradiol elevated the expression of hnRNPA1 at the gene level (Figure 1A) and inhibited cell growth (Figure 1B) in both cell lines. The Hacat cells were much less sensitive to the 10×10^-13^M estradiol compared to that of melanoma GLL19 cells. Therefore, we selected 10×10^-13^M estradiol to treat cells in the following experiments.

We next investigated the effects of estradiol on the expression and cellular function of hnRNPA1 and MDM2. 10×10^-13^M estradiol induced the expression of hnRNPA1 at mRNA (Figure 1C) and protein level (Figure 1D) in Hacat, GLL19 and A375 cell lines. In the meanwhile, MDM2 (Figure 1C, 1D) were negatively regulated after the treatment of estradiol. For GLL19 and A375 cell lines, the staining for hnRNPA1 were significantly elevated in the estradiol-treated cells in comparison with control cells (Figure 1E). But there was no change about the location of hnRNPA1. These results suggested that estradiol, which influenced the MDM2 expression in skin cancer cells, elevated the expression of splicing factor hnRNPA1 but did not alter its location.

Considering that the MDM2 gene is correlated to P53, the potential roles of P53 in the decline of MDM2 with estradiol-treatment were investigated. After the treatment with estradiol for 96 hours, the Hacat, GLL19 and A375 cell lines were harvested and P53 expression was measured at mRNA level (Figure 1C). In addition, the modification of P53 expression at protein level was determined by western blot (Figure 1D), and there was just a slight change in P53 after the treatment of estradiol. Consequently, the decrease of MDM2 by hnRNPA1 did not correlate with a rise in P53.

The basics of RIP are very similar to chromatin immunoprecipitation, and following immunoprecipitation of a protein association with specific nucleic acid regions were identified by RT-PCR. What's more, RIP can provide snapshots of protein-RNA interactions at specific time points and hence is useful for kinetic analyses of events occurring on RNA *in vivo* [29, 30]. Additionally, we collected the same number of cells between control and treatment group, and then assay hnRNPA1-MDM2 binding through RIP. HnRNPA1 protein was immunoprecipitated with its associated MDM2 pre-mRNA, which wasdetected by RT-PCR (Figure 1F), demonstrating that hnRNPA1 can bind to mdm2 pre-mRNA directly in GLL19 and A375 cell lines.

### Estradiol suppressed the proliferation and migration of melanomas *in vitro*

**Figure 2:**
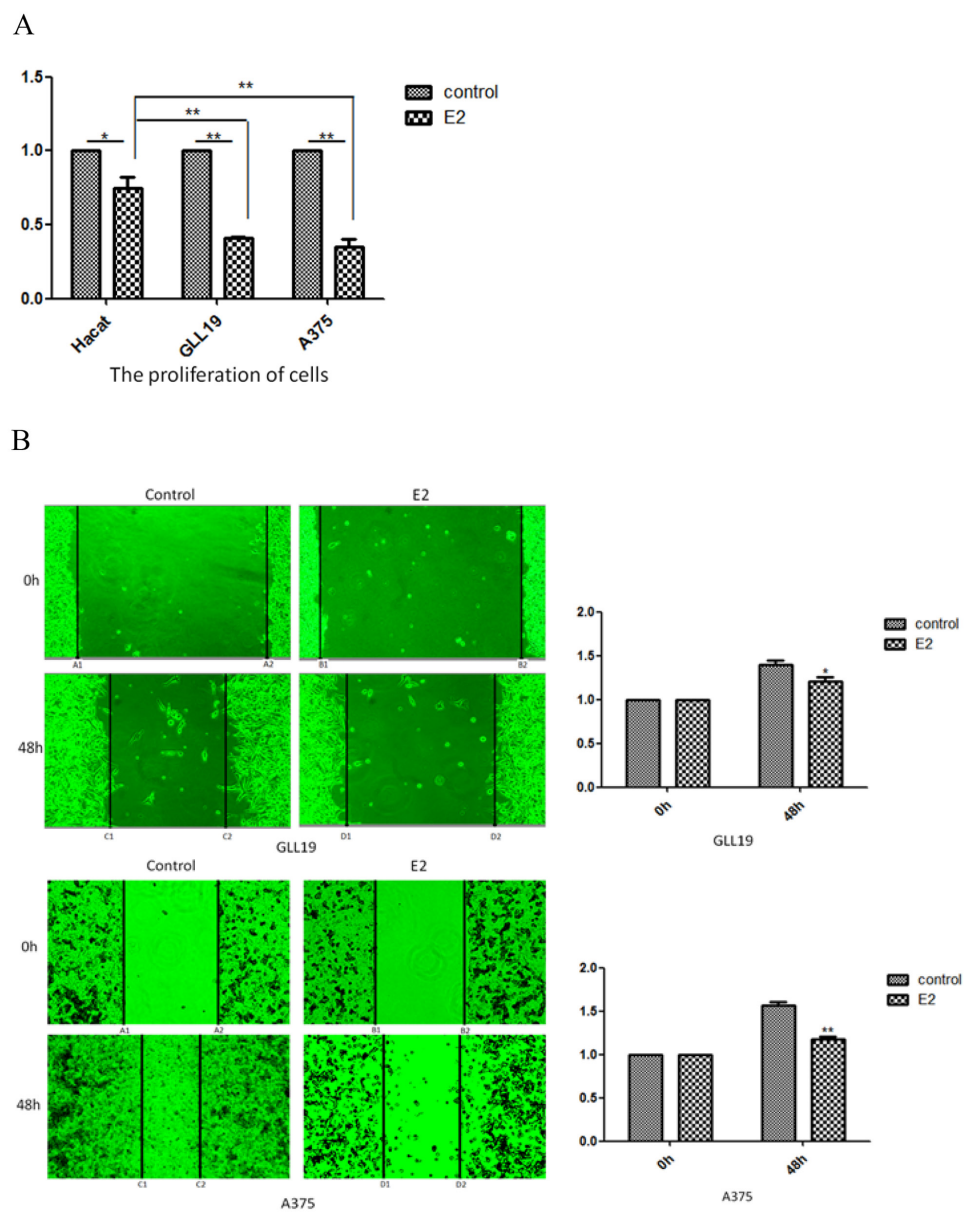
The proliferation and immigration of cells were inhibited by estradiol treatment **A.** Hacat, GLL19 and A375 cells were exposed to 10×10-13M estradiol for 96 hours, and MTT assay was used to test the change of cell proliferation. **B.** Confluent monolayer of GLL19 and A375 cell lines were wounded (A1A2 and B1B2) and cultured with mitomycin, 10×10-13M estradiol and vehicle. Estradiol treatment induced the decrease of migration areas (“A1C1 plus A2C2”vs “B1D1 plus B2D2”) as compared to thevehicle-treated cells.(n=3, a representative experiment is shown; ‘*’means P<0.05; ‘**’means P<0.01).

To demonstrate the anticancer activity of estradiol, Hacat, GLL19 and A375 cells were treated with 10×10^-13^M estradiol for 96 hours. MTT assay was used to determine the effect of estradiol on cell proliferation. As shown in Figure 2A, the estradiol resulted in approximately 60% inhibition efficiency on cell proliferation in the GLL19 and A375 cells, and the effects were more markedly (P<0.01) than that in Hacat cells which are immortalized but not malignant keratinocyte, indicating that the decrease of MDM2 were critical for the estradiol-induced proliferation inhibition in skin cancer cells. Migration was studied using a scratch assay after theincubation of estradiol or vehicle. Estradiol treatment induced 39% and 19% decrease of migration areas as compared to vehicle-treated in GLL19 and A375 cell lines, respectively (Figure 2B). Collectively, estradiol acted as negative regulator for the proliferation and migration of melanoma.

### Estradiol induced the changes of marker genes in epithelial-mesenchymal transition

**Figure 3:**
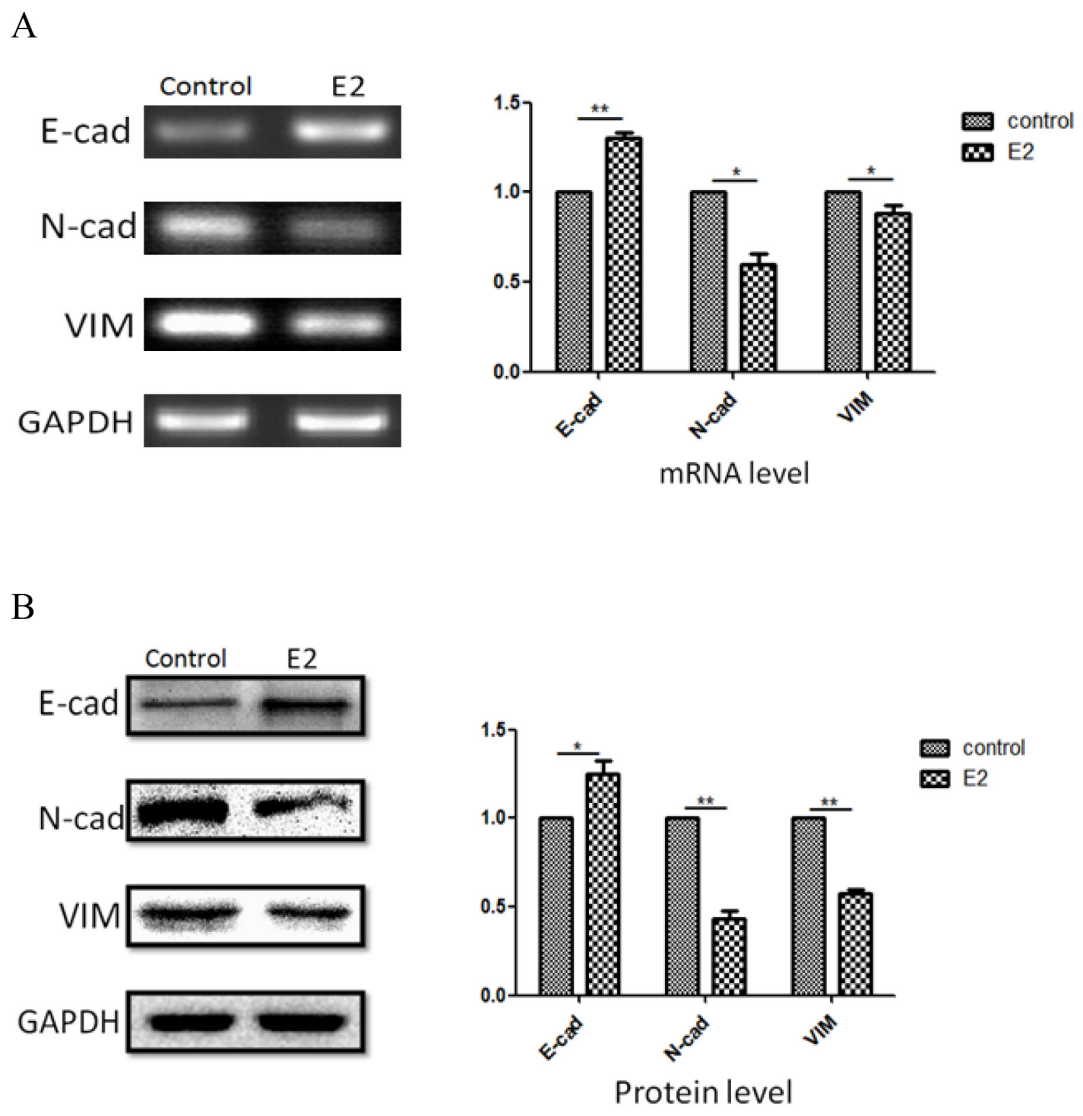
Correlations of estradiol on expressions of E-cadherin, N-cadherin and vimentin A375 cancer cells were incubated with 10×10-13M estradiol for 96 hours, and expression of these three markers were measuredat gene level **A.** and protein level **B.** Results were expressed as percent of GAPDH vs. vehicle-treated cells. (n=3, a representative experiment is shown; ‘*’means P<0.05; ‘**’means P<0.01).

It is well known that epithelial-mesenchymal transition (EMT) is necessary for theprogression of several kinds of cancers, including down-regulating the expression of epithelial marker such as N-cadherin, and up-regulating the expression of mesenchymal markers such as E-cadherin and vimentin [31]. Results showed that estradiol progressively increased E-cadherin expression which is epithelial cell marker almost 35% after 96 hours; while, estradiol decreased N-cadherin and vimentin levels, which are mesenchymal cell marker significantly (P<0.01) (Figure 3).

### Depletion of hnRNPA1 expression increased the MDM 2 expression

**Figure 4:**
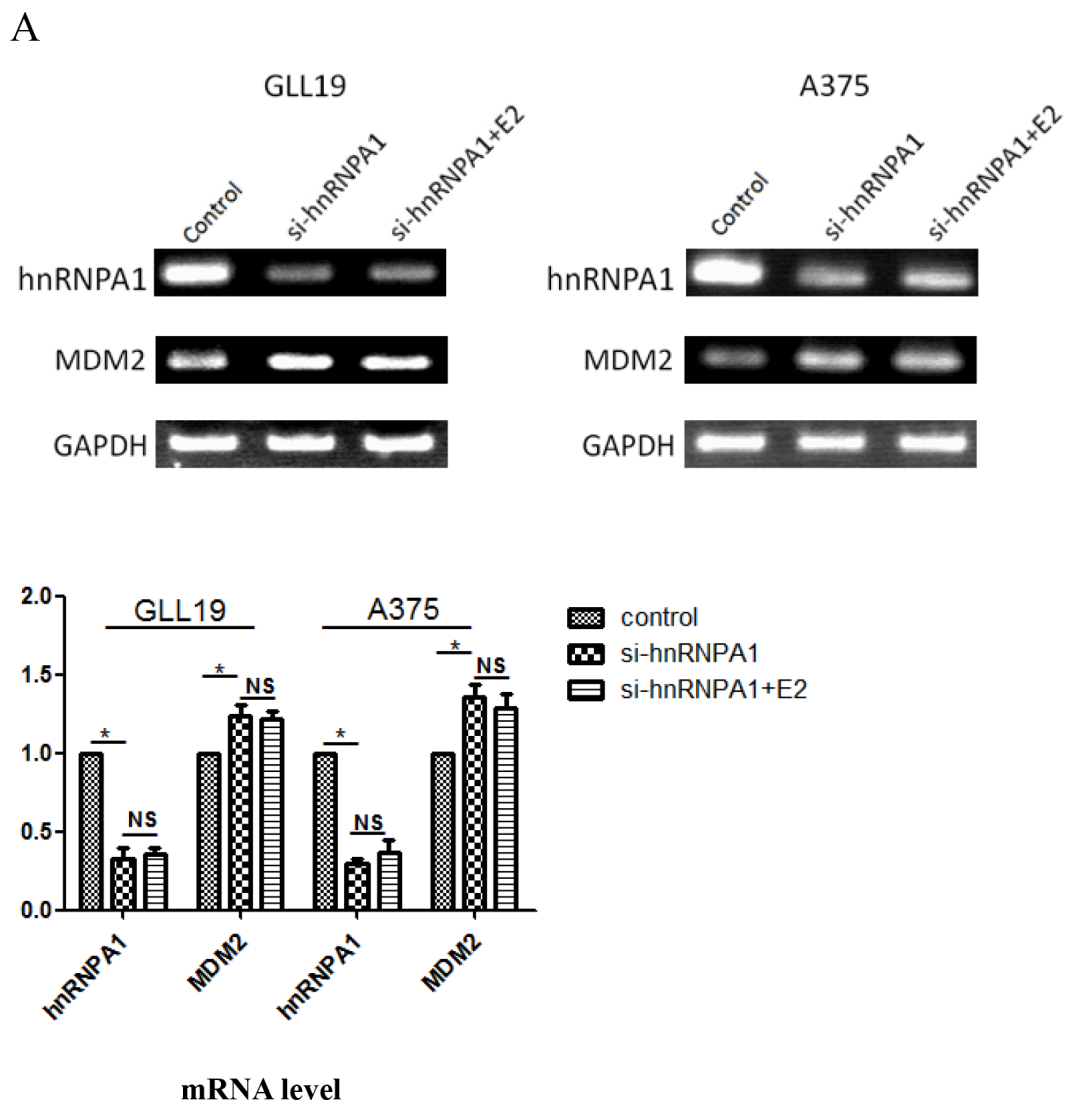
Changes of the MDM2 expression by depleting of hnRNPA1 GLL19 cells were **A.** further investigation by transfected with control siRNA and hnRNPA1 siRNA±estradiol, then the relative mRNA level or **B.** protein level of hnRNPA1 as well as MDM2 were presented by the quantitative values on these charts. Results were expressed as percent of GAPDH vs. vehicle-treated cells. (n=3, a representative experiment is shown; ‘*’means P<0.05; ‘**’means P<0.01. ‘NS’ means no significance).

We have found that MDM2 was negatively regulated by the up-regulation of hnRNPA1. Next, we determined whether or not depleting the expression of hnRNPA1 can up-regulate the MDM2 mRNA expression. We further investigated the effects of hnRNPA1 knockdown on MDM2 expression in GLL19 and A375 cells by RT-PCR (Figure 4A) and western blot (Figure 4B). Results indicated that silence of hnRNPA1 led to the increase of MDM2 at protein levels, which can be observed obviously in melanoma cells (P<0.05).

### hnRNPA1 declined MDM2 expression may not only relate to the estrogen receptor

**Figure 5:**
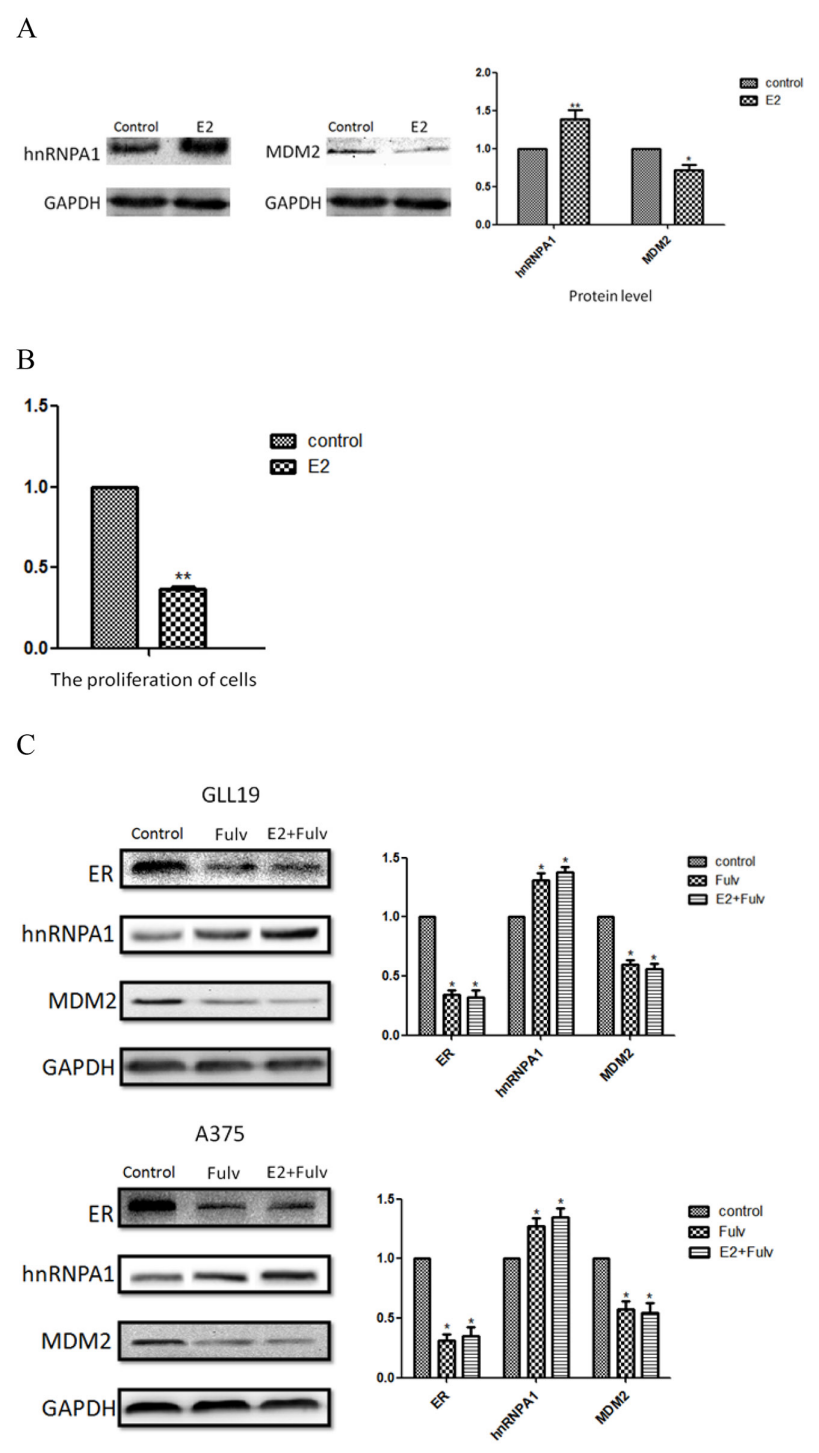
The ER is not a critical factor in the regulation of MDM2 by hnRNPA1 MDA-MB-231 cells were treated with or without 10×10-13M estradiol for 96 hours before evaluating the hnRNPA1 and MDM2 expression **A.** at protein level. **B.** MTT assay was used to test the change of cell proliferation. **C.** ER, expressions of hnRNPA1 and MDM2 were detected at protein levels after MDA-MB-231 cells were cultured in the 50uM fulvestrant with or without estradiol for 96h. Results were expressed as percent of GAPDH vs. vehicle-treated cells. (n=3, a representative experiment is shown; ‘*’means P<0.05; ‘**’means P<0.01).

GLL19 and A375 are highly metastatic melanomas that can express estrogen receptors [12, 32]. Considering that the transcriptional activation of MDM2 canrespond to estrogen through estrogen receptor (ER), we further investigated the influence of estradiol on MDA-MB-231 cellswhich are estrogen receptor negative (ER-) cells [33]. These data were similar to those in GLL19 and A375 cells and we confirmed that estradiol also up-regulated hnRNPA1 and then altered the expression of MDM2 (Figure 5A). The estradiol resulted in approximately 60% inhibition efficiency on cell proliferation in the MDA-MB-231 cells (Figure 5B) (P<0.01). In the next section, we used fulvestrant which acts as an ER antagonist to suppress the expression of ER in GLL19 and A375 cells, following with the treatment of estradiol as before [34]. It was interesting to find that fulvestrant leading to the up-regulation of hnRNPA1 at the same time, and the results almost had no differences after the addition of estradiol (Figure 5C). These experiments imply that hnRNPA1 correlating MDM2 expression may not only relate to the estrogen receptor.

### Estradiol inhibited tumor growth and reduced MDM2 expression *in vivo*

**Figure 6:**
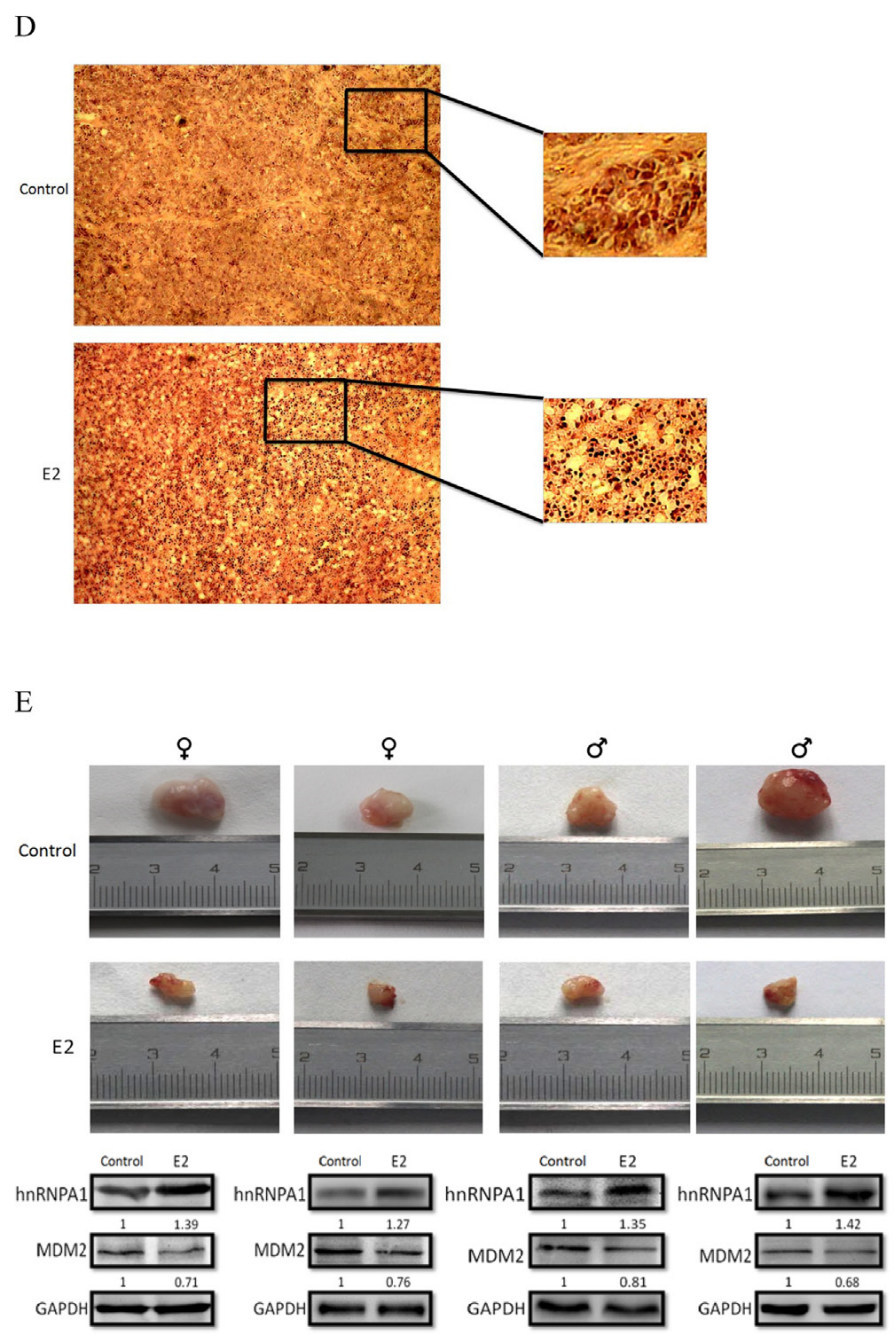
In vivo evaluations In order to know inhibition of estradiol on nude mice bearing melanoma in vivo, estradiol was administered by subcutaneous injection every two days near the tumor at doses of 2.5mg/kg/d for about 16 days. At last, all the tumors were removed and analyzed. The volume **A.** and weightofeach tumor **B.** or mouse **C.** were measured periodically. **D.** Moreover, some parts of melanomas were embedded with paraffin and sliced with H&E in order to observe the damage of estradiol to tumors. **E.** Finally, to validate the mechanisms of estradiol in vivo, we assessed the expression of hnRNPA1 and MDM2 of tumor tissues at protein levels in vitro. Results were expressed as percent of GAPDH vs. vehicle-treated cells. (n=4, a representative experiment is shown; ‘*’means P<0.05; ‘**’means P<0.01).

To verify the antitumor activity for estradiol for clinical application *in vivo*, we established several tumor models through subcutaneously inoculated A375 cells into nude mice. Then we divided them into two groups (n=4, female=2, male=2) randomly. Mice in one group were treated with alcohol (control), and mice in the other group were treated by intraperitoneal injections of estradiol (2.5mg/kg/d). Visual results indicated that the estradiol suppressed the growth of melanoma (Figure 6A, 6B, 6E). On day 16 of estradiol treatment, the volumes of tumor in the treated mice were decreased by about 75% compared with that in vehicle treated group (P <0.01). We also measured the weight of each mouse periodically, and found that there were slight descend on body weight after the treatment of estradiol every two days (Figure 6C). Moreover, all melanomas were removed from tumor models, and then some parts of them were embedded with paraffin and sliced with hematoxylin and eosin (H&E). The sliced sections were observed and showed that estradiol caused damage to tumor tissue compared with the control samples (Figure 6D). From the picture, we can find that estradiol will damage tissues via fiber breakage with a large section of cell shrinkage [35, 36].

To validate the mechanisms of estradiol *in vivo*, we assessed the expression of hnRNPA1 and MDM2 of tumor tissues at protein levels (Figure 6E). Consistent with the results of our *in vitro* experiments, the expression of hnRNPA1 was increased and the expression of MDM2 was decreased.

## DISCUSSION

Until now, the activation or inhibition of estradiol on tumors were inconsistent in a lot of researches. Several experiments showed that estradiol can inhibit the growth of tumor. For example, estradiol could induce apoptosis through activation of P53 in liver cancer development, and a new kind of estradiol-cationic lipid hybrids which has ten-carbon twin chain exhibitedabetter anticancer activity than estradiol [11, 37]. The estradiol had significant cytotoxicity against CHO and NIH 3T3 (two kind of non-cancercells), and it also induced cell apoptosis in MIAPaCa-2 and B16F10 (two kinds of cancer cells) [11, 12]. On the contrary, many other researchers revealed that estradiol contributed to the activity of tumors. For the suppression of estradiol on cell proliferation, some experiments proved that the proliferation of MCF7 cells was stimulated by estradiol through a dose-dependent way; the proliferative of wild-type MCF7 cells responded to 100nM estradiol in the first 3~5 days; the number of mouse melanoma cell line B16K1, mouse lung carcinoma cell line LL2 and mouse breast tumor cells 4T1 were up-regulated *in vivo* with estradiol-treatment [38–41]. Our outcome in accordance with the former opinion: estradiol prevented the proliferation and migration of GLL19 and A375 cells, and because these two kinds of cell lines are human melanoma, we got a high level of similarity of results between GLL19 and A375. The estradiol acted as a negative regulator for epithelial-mesenchymal transition progression of skin cancer cells.

The effect of estradiol on tumor growth may come from different pathways. For instance, estradiol could increase P53 and P21 expression in Ch27 and H1355 which are lung cancer cell lines [5]. However, in ER+ skin cancer cells, anticancer correlationswere caused by preventingnitric-oxidesynthase through estrogen receptors, and estradiol incited the level of estrogen receptors [12]. Moreover, estradiol as pharmacophores could conjugate with numerous cationic lipids and then led to the apoptosis via generation of Reactive Oxygen Species, activation of protein kinase C and mitogen-activated protein kinase, modulation of caspases, etc [42–44]. Interestingly, although MDM2 was decreased by estradiol-induced hnRNPA1, there was just a slight change of P53 expression after estradiol-treatment in our study. We inferred that the estradiol did not alter levels of P53. Therefore, the effect of estradiol on MDM2expression provides a novel mechanism that MDM2 inhibits the tumor progression may not correlate to P53. Additional analyses *in vivo* after injection of estradiol in nude mice further verified the inhibitive effect of estradiol on the growth of melanoma. These results corroborated the stand that estradiol was performed as an anti-skin cancer agent *in vivo* and *in vitro*.

It is well known that the MDM2 performs its oncogenic effects through p53-dependent and p53-independen ways, which controls the cell cycle, contributes to the genomic integrity of different cancer cells, and influences the response to numerous DNA damage [45–47]. In 1996, multiple-sized MDM2 transcripts were identified from tumor samples in the lab of Lunec and named from MDM2a to MDM2e [48, 49]. Variants of MDM2 show different effects on P53 compared to the wild type MDM2, indicating that MDM2 alternative splicing was critical to the action of the tumor suppressor P53 [50]. For example, MDM2^alt1^(also named MDM2b )one of the common variants of MDM2, has been detected to be over-expressed in a lot of tumors [51]. MDM2^alt1^not only lacks the binding domain with P53, but also declines the expression of MDM2. Thus this kind of variant can act as a negative modulator of MDM2-P53 interaction [52–54]. Therefore, decrease of the expression of wild type MDM2via critical splicing factors is a promising method to tumor inhibition. In our experiments, we proved the hnRNPA1-MDM2 binding through RIP, and found that estradiol-inducedsplicing factorhnRNPA1 resulted in decreased MDM2, showing the negative correlation betweenhnRNPA1 and MDM2. Meanwhile, a dose-dependent stimulation of hnRNPA1 was observed with concentration ranging from 10×10^-9^to10×10^-13^M estradiol. At last, the concentration of estradiol which we chose was 10×10^-13^M because it was beneficial to hnRNPA1 expression in melanoma. Although MDM2 is an oncogene that is overexpressed in various types of cancers, it is also expressed in Hacat in a lower level, which makes the Hacat cells were much less sensitive to estradiol(10×10^-13^M) compared to that of GLL19 and A375 cell lines. Conversely, the depletion of hnRNPA1 through transfection of hnRNPA1 siRNA led to the increase of MDM2 at both protein level and gene level. It is more interesting that although estradiol always functions via estrogen receptor, the estradiol can mediate hnRNPA1 level may not only relate the estrogen receptor in GLL19 and A375 cell lines. Of note, the estradiol has been widely used in the researches of breast cancer cells and the estrogen-sensitive gene expression [13, 15]. In addition, Sayantani unfolded estradiol has become one of a stimulus of melanoma’ apoptosis via the function of estrogen-linked L-nitro-arginine molecule [12]. But the report about mechanisms of hnRNPA1 regulating MDM2 expression in skin cancer cells is poorly. Our data predicted that the estradiol stimulate tumor apoptosis by elevating the level of hnRNPA1, and then influenced MDM2 expression in different types of cell lines. The phenomena associated with the melanoma cell killing bestowed that the estradiol has an anticancer property.

As pointed out by our experiment *in vivo*, we showed that estradiol-treatment rendered melanoma tumors into a less aggressive status. Consistent with the experiment *in vitro*, estradiol reduced MDM2 expression through elevating the level of hnRNPA1 in A375 tumor tissue. In addition, *in vivo* study, control mice with growing tumors gained weight between day0 and day10 and kept that weight until the end of the experiment. Mice treated with estradiol lose tumor weights between day2 and day8. We speculated that tumor growth was restrained in treat-group mice, but estradiol may promote the metabolism of mice, and speed up the energy consumption.

In summary, the estradiol is a rationally anti-skin cancer agent that induces MDM2 down-regulation via enhanced hnRNPA1 expression, and controls the oncogenic activities of MDM2 in melanomas *in vitro*/*vivo* testing. These findings would help provide a basis to investigate the therapeutic potential for future preclinical or clinical development of estradiol which focus on MDM2 itself underlining molecular mechanisms.

## MATERIALS AND METHODS

### *In Vitro* evaluations

### Cell lines and culture

Hacat (one kind of immortalized but nonmalignant keratinocyte), human melanoma cell lines GLL19 and A375 were acquired from Dr. Zhong (Chongqing University, China). MDA-MB-231 human breast cancer cells were supplied by Dr. Chen (Chongqing Medical University, China). Hacat, GLL19 and MDA-MB-231 cell lines were maintained in RPMI 1640 medium (Hyclone, Utah, USA), and A375 cell line were maintained inDMEM high glucose medium (Gibco, Grand Island, NY). These media were supplemented with 10% fetal bovine serum (ExCell Bio, China), 100 units/ml penicillin (Beyotime, China) and 100 ug/ml streptomycin (Beyotime, China) in a humidified atmosphere which containing 5% carbon dioxide/95% air at 37°C.

### Semi-quantitative PCR

Total RNA was extracted using the TRIzol reagent (Invitrogen, USA) according to the manufacturer's protocol. The first strand cDNA was reverse-transcribed from total RNA using the PrimeScript^TM^ RT reagent Kit (TaKaRa, Japan). The primers for glyceraldehyde-3-phosphate dehydrogenase (GAPDH) were: 5′-GGAGCGAGATCCCTCCAAAAT-3′ (GAPDH-F), 5′-GGCTGTTGTCATACTTCTCATGG-3′(GAPDH-R). The primers detecting mdm2 were: 5’-GAATCATCGG ACTCAGGTACATC-3’ (MD- M2-F), 5′-CTTTGTCTTGG GTTTCTTCC-3′(MDM2^alt1^-R). The primers of hnRNPA1 were 5’-TACGTTCGTCAGCTTGCTCC-3’ (hnRNPA1 -F), 5’-TCATTACCACACAGTCCGTGA-3’(hnRNPA1-R). These results were normalized against GAPDH as an endogenous control. The DNA polymerases were purchased from Tiangen (China). The PCR parameters were set as 95 °C for 5 min, followed by 40 cycles of 95 °C for 30 s, 53.5 °C for 30 s and 72 °C for 50 s.

### Western blot analysis

The cells were lysed in RIPA lysis buffer (50 mM Tris-Cl pH 7.5, 1% NP40, 0.5 mM EDTA, 0.1% SDS, 150 mM NaCl, 0.5% Sodium deoxycholate) (Bioteke, China) with PMSF (Beyotime, China). Lysates were clarified by centrifugation at 14,000 rpm for 10 min at 4°C. Protein concentration was measured by BCA assay (Beyotime, China). The protein samples were separated onto 15% SDS-PAGE gels, and then transferred to polyvinylidene fluoride (PVDF) membranes (PALL, USA). Membranes were blocked in TBST with 5% nonfat powered milk and then incubated with primary antibodies at 4°C overnight. After washing with TBST, the membranes were incubated with corresponding secondary antibodies at room temperature for 1h. Following a triple washing step with TBST, the bands were visualized with the enzyme-linked chemiluminescence method (Millipore, USA). Primary antibodies including GAPDH (Proteinch, China), hnRNPA1 (Proteinch, China), MDM2 (BBI Life Science, China), P53 (Proteinch, China), ESR1(BBI Life Science, China), Vimentin (Proteinch, China), E-cadherin (Santa Cruz, USA), N-cadherin (Santa Cruz, USA) and HRP-conjugated anti-rabbit or anti- mouse secondary antibodies. Quantification of protein bands was performed using Quantity One software.

### Drug treatment for cells

Different kinds of cells treated with vehicle (DMSO) or 10×10^-13^M estradiol (Melonepharma, China) for 96h or 50uM fulvestrant (Selleckchem, USA) for 96h. All of these drugs were stored at -20°C, and then diluted in media when they were needed in medium. All the cell extracts from these treated cells were collected at the indicated times and then analyzed by(3-4,5-dimethyl-2-thiazolyl)-2,5-diphenyl-2-H-tetrazolium bromide (MTT) assay (Dingguo, China)which through analyzed the absorbance of each treated and untreated sample, Semi-quantitative PCR and Western blotting.

### SiRNA transfection

The sequence of si-hnRNPA1 which was synthesized at GenePharma (China) was 5’-CAGCUGA GGAAGCUCUUCA-3’. The si-hnRNPA1 was transfected into different kinds of cells using X-tremeGENEsiRNA Transfection Reagent (Roche, USA) following the manufacturer's instructions. The results were determined by semi-quantitative RT-PCR or western blot.

### Cytotoxicity assay

Different kinds of cells were seeded into the 96-well plate at 3000 cells/well and cultured overnight, then treated with different drug combinations: 10×10^-13^M estradiol treated on cells for 96h. The cell proliferation was measured as a percentage of control by MTT assay. In brief, 150 ml MTT solution at a final concentration of 0.5mg/ml was added to each well, and the cells were incubated with MTT for 4 hours at 37°C. Next, the solutions of MTT were discarded and 150 ml DMSO was added to each well. After dissolve the crystals by the shaker for 20 minutes, we used microplate reader (Bio-Rad, Germany) to measure the absorbance at the wavelength of 540 nm.

### Migration assay

Confluent monolayer of GLL19 and A375 cell lines were wounded with a scratchand cultured with mitomycin which can inhibit the proliferation of cells so that cells closed the gap were due to migration instead of proliferation. At the same time, let the wounded cell lines incubated in estradiol or vehicle for 48 h. The migration areas were monitored using microscope and Charge Coupled Device with the ImageJ analysis software.

### Immunofluorescence assay

Treated GLL19 and A375 cells were fixed with 4% paraformaldehyde in PBS for 45min. GLL19 and A375 cells were permeabilized with 0.3% Triton-X 100 and blocked with 2% BSA in PBS for 30min and 20min, respectively. Subsequently, samples were incubated with primary antibody of hnRNPA1 (1:200) in 4°C atmosphere overnight. The corresponding secondary antibody was fluorescein isothiocyanate (FITC)-conjugated (Beyotime, China) anti-rabbit (1:200). Nuclei were counterstained with 1 μg/mL bisBenzimide H 33258 (Hoechst33258) (Beyotime, China). Images were obtained using fluorescence microscope and Charge Coupled Device.

### RNA immunoprecipitation

RNA immunoprecipitation is an antibody-based method by which specific proteins can be immunoprecipitated with its bounded RNA and the results are identified by RT-PCR [55–57]. A375 and GLL19 cells were seeded in a 10cm cell culture dishes, and cells were harvested and re-suspend in PBS when reaching to 70-80% confluent in dishes. After centrifugation at 3000rmp for 3min, cells were re-suspended in RIPA lysis buffer (50 mMTris-cl pH 7.5, 1% NP40, 0.5mM EDTA, 0.1% SDS, 150mM NaCl, 0.5% Sodium deoxycholate). Next, hnRNPA1 antibody was added into the supernatant and incubated overnight at 4°C. After that, the protein A/G beads (Beyotime, China) were incubated with rotation at 4°C for 1 hour. All the material which were unbound to A/G beads were washed by RIP buffer (150mM KCl, 25mM Tris PH 7.4, 5mM EDTA, 0.5mM DTT, 0.5% NP40, 100U/ml RNAase inhibitor, protease inhibitors) for three times. Beads were re-suspended in Trizol reagent for RNA isolated. Following the RNA were transcribed into cDNA, the results were obtained by PCR.

### *In Vivo* evaluations

### Establishment of tumor model

All *in vivo* experiments on nude mice was designed and performed according to the guidelines which come from the Institutional Animal Care and Use Committee of China. Eight female and male nude mice about 5 weeks old were bought from the Animal Laboratory in Xinqiao Hospital (Chongqing, China). The tumor models were established on naked mouse via subcutaneously injecting 100μL of PBS (pH7.2) containing 2 × 10^7^ A375 cells at the groin side of each female and male nude mouse.

### In vivo tumor treatment with estradiol

When the size of tumor reached about 50 mm^3^, these tumor-bearing nude mice were randomly divided into two groups which have similar weight and tumor size. Estradiol was subcutaneous injected near the tumor of mice; while the mice in the control group were only treated with alcohol. The estradiol amount defined as 2.5mg/kg/d. Mice were administrated every two days. Using a caliper to record size of tumor, the volume of tumor was calculated as following equation: V_tumor_ = ab^2^/2 (‘a’ means the maximum length of tumor; ‘b’ means the minimum width of tumor). The volume of tumor and body weight of each mouse was recorded and calculated before each administration. The mice were sacrificed after 16 days of subcutaneous injection and the tumor tissues were taken out from these mice for further detections.

### Examination for tumor tissues

Divided each tumor into two part—one for histological assay and the other one for Western blot analyses. The parts of tumor for histological examination were fixed in formalin solution (10%) at 4 °C for 48 h, and then embedded with paraffin and sliced for hematoxylin and eosin (H&E) stain. The stained sections were observed and photographed using an optical microscope and Charge Coupled Device.

### Statistical analysis

Each experiment was done at least three independent times. We presented all quantitative data as the means±standard deviation in the current study. The assay data were analyzed using a 2-tailed analysis of variance or Student's t-test, and P values which were less than 0.05 can be regarded as statistically significant differences.

Performed data analysis: Li Li, Jianguo Feng, Liling Tang;

Participated in research design: Li Li, Jianguo Feng, Liling Tang;

Conducted experiments: Li Li, JianguoFeng, Ying Chen, Shun Li, MengtingOu, Weichao Sun.

## Abbreviations

EMT: epithelial-mesenchymal transition
ER: estrogen receptor
hnRNPA: arginine/serine-rich proteins and heterogeneous nuclear ribonucleo protein
MDM2: mouse double minute
